# Supplementary material for: Optogenetic cleavage of the Miro GTPase reveals the direct consequences of real-time loss of function in Drosophila
Source: PLoS Biol. 2023 Aug 17;21(8):e3002273. doi: 10.1371/journal.pbio.3002273 (PMC10465005; doi:10.1371/journal.pbio.3002273)
Supplement: S1 Raw Images — In the top panel in (A), the faint double bands between the 102 and 150 molecular weight markers indicate nonspecific binding of the Miro antibody. In the middle panel in (D), the bands of approximately 76 kDa correspond to endogenous Miro. The absence of endogenous Miro in the “IP” lanes indicate that transfected Split-Miro does not bind to endogenous Miro at high affinity. Panels in “Supporting Figure 5” include original blots used in S5(A) Fig. The band above the 150 molecular weight marker indicates nonspecific binding of the Miro antibody. In all panels, “X” above a lane indicate that lane is not included in the final figure. (PDF) [file pbio.3002273.s001.pdf]

Supporting Figure 2

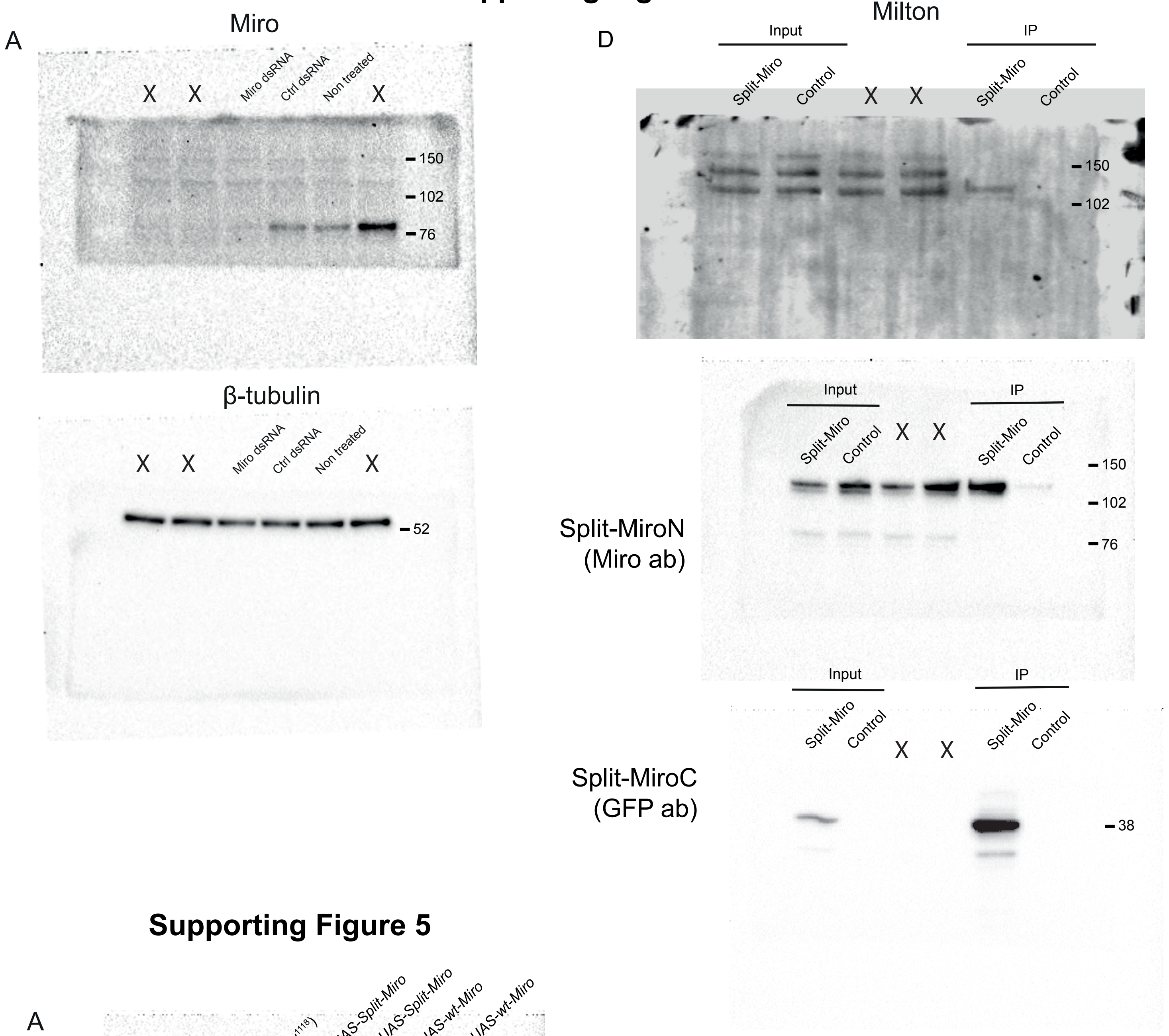

Supporting Figure 5

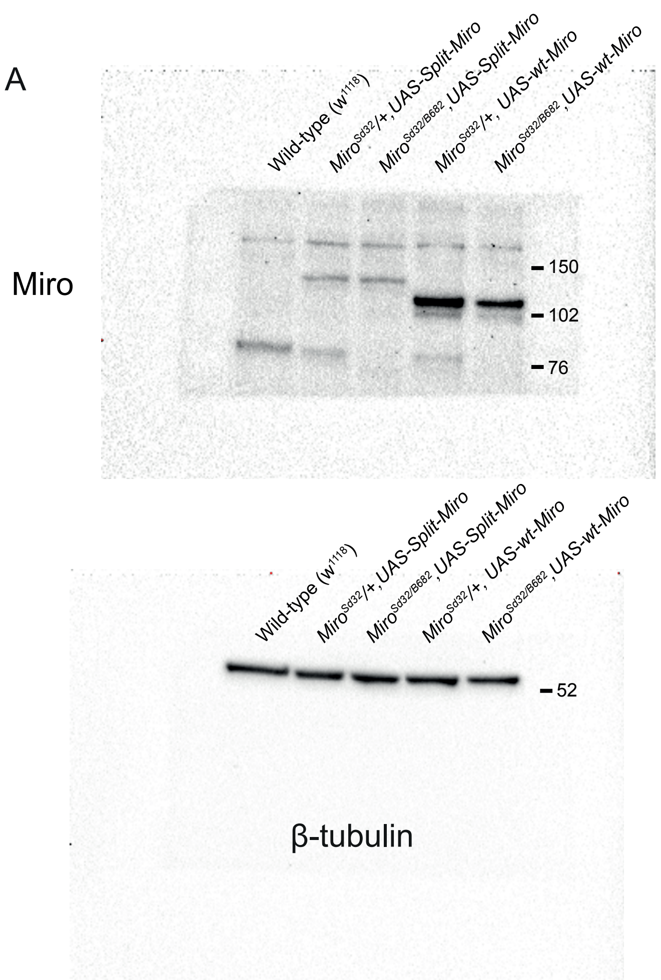

Images were acquired with ChemiDoc, except for top panel in Supplementary Figure 2D (Milton) where LI-COR Odyssey was used.
