# Supplementary figures and images for: Optogenetic cleavage of the Miro GTPase reveals the direct consequences of real-time loss of function in Drosophila
Source: PLoS Biol. 2023 Aug 17;21(8):e3002273. doi: 10.1371/journal.pbio.3002273 (PMC10465005; doi:10.1371/journal.pbio.3002273)

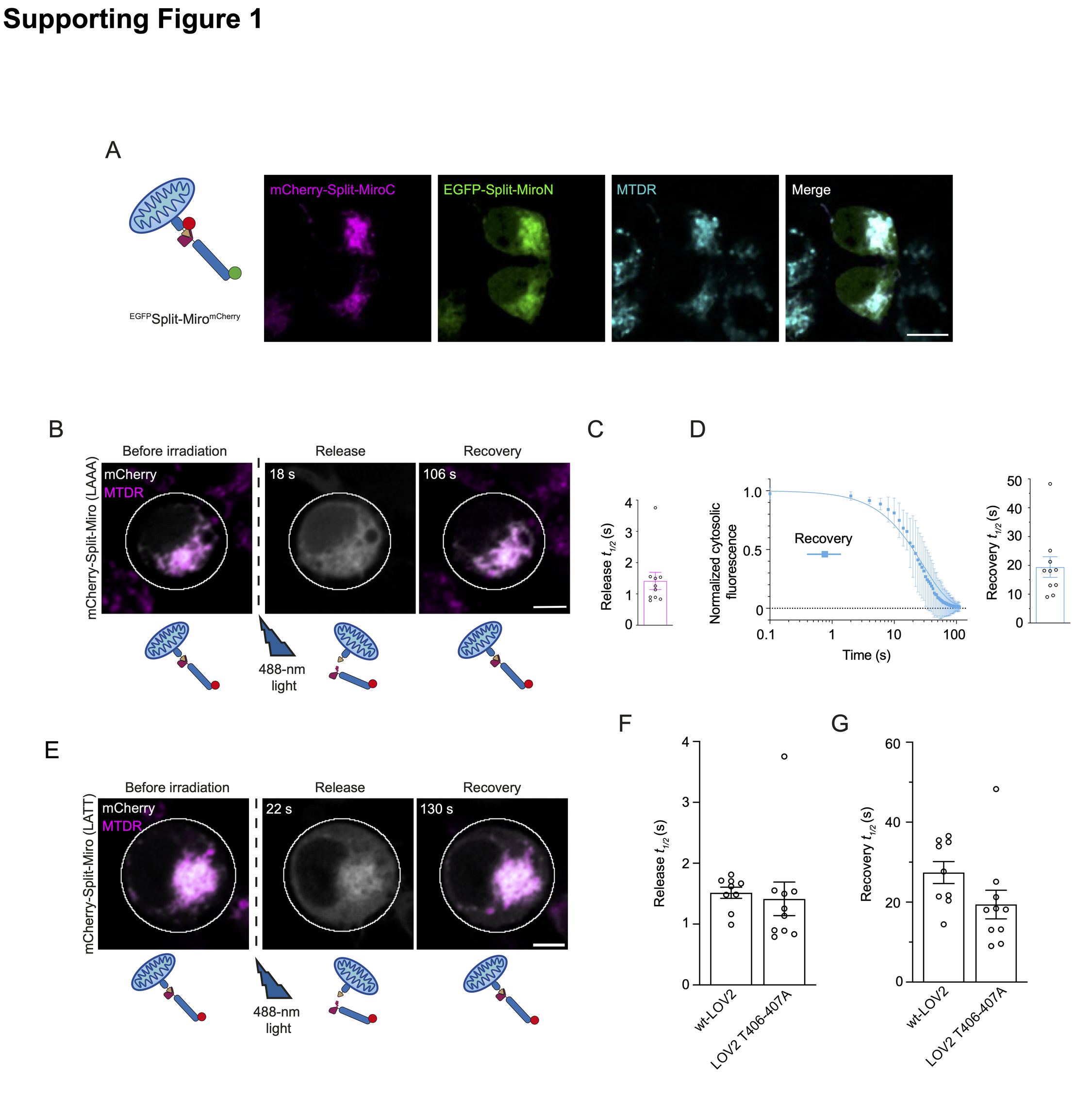

Supplement: S1 Fig — (A) EGFP-Split-Miro-mCherry is reconstituted and localises at the mitochondria in the absence of sustained blue light irradiation. S2R+ cells were cotransfected with mCherry-tagged Split-MiroC (magenta) and EGFP-tagged Split-MiroN (green), mitochondria are stained with MitoTracker Deep Red (MTDR, cyan). Note the EGFP/mCherry tags are appended at different termini compared to the mCherry-Split-Miro-EGFP shown in Fig 1. (B, E) Localisation of mCherry-Split-MiroN before and after a 570-ms pulse of blue light. S2R+ cells are cotransfected with untagged Split-MiroC and mCherry-Split-MiroN containing the T406A, T407A mutations in the N-terminus of LOV2 (LAAA, in B) or mCherry-Split-MiroN containing the wild-type sequence (LATT, in E). Before irradiation, Split-Miro is reconstituted at the mitochondria, indicated by the colocalisation of mCherry-Split-MiroN (grey) and the MitoTracker Deep Red (MTDR) staining. Immediately after irradiation (18 seconds), Split-MiroN is released into the cytoplasm (indicated by a more homogenous grey colour), and it fully reconstitutes within 2 minutes. (C, D) Quantification of mCherry-Split-MiroN half-time release (C) and recovery (D) after photocleavage, relative to (B). In (D), left panel shows levels of cytosolic Split-Miro N-terminus quantified after the maximum release is reached; right panel: recovery half-time. Solid line in (D) is exponential curve fit. In (B) and (E), the cartoon depicts photocleavage and reconstitution of mCherry-tagged Split-Miro. s, seconds. Scale bar: 10 μm. (F, G) Comparison of mCherry-Split-MiroN release (F) and recovery (G) half-time after photocleavage shows no significant difference between the 2 LOV2 variants (unpaired Student’s t test). The LATT (wild-type) to LAAA mutation in the N-terminus of LOV2 has been reported to have a stabilising effect on the Jα helix in cultured cells at 37°C [17,18]. We did not find any noticeable difference in the steady-state reconstitution efficiency of the 2 variants in S2 [file pbio.3002273.s003.tif]

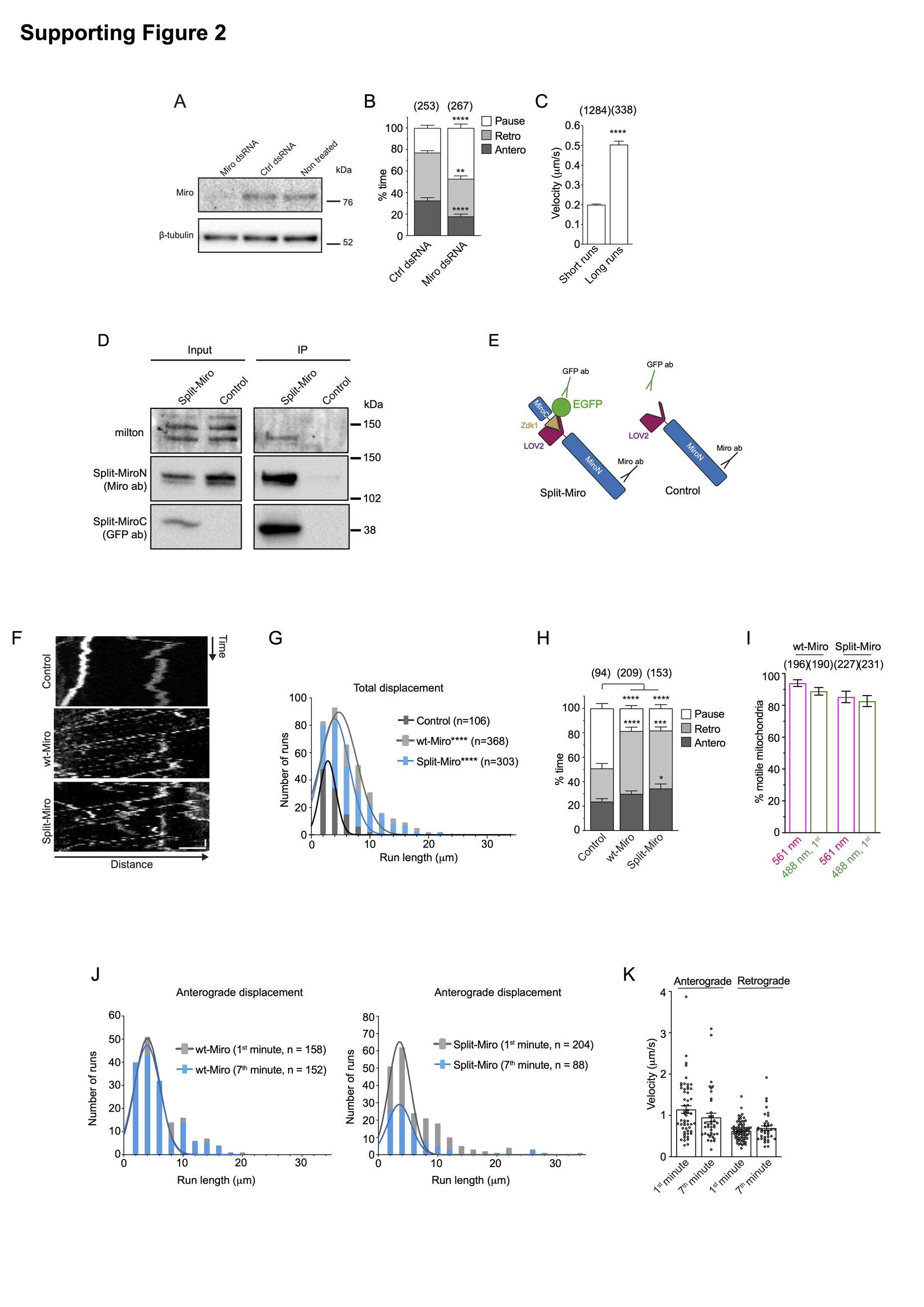

Supplement: S2 Fig — (A) Representative western blots of Miro from total lysates of control and Miro RNAi-treated S2R+ cells. (B) Duty cycle analysis describes the average time mitochondria spend moving anterogradely, retrogradely, or pausing. For each parameter, all mitochondrial values from each cell were averaged and compared between control and Miro dsRNA condition using a multiple Student’s t tests. Number of mitochondria analysed are in brackets from 29 (Ctrl dsRNA) and 36 (Miro dsRNA) cells, respectively, from 2 independent experiments. (C) Run velocities of short and long runs in control dsRNA-treated S2R+ cells showing that Miro-dependent long runs are significantly more processive than the short, Miro-independent runs (Fig 2C). Number of runs analysed are in brackets, from 2 independent experiments. Mann–Whitney test. (D) Split-Miro interacts with Milton in S2R+ cells. Cells were transfected with Split-Miro or Control (as shown in E) and the total cell lysates immunoprecipitated using GFP-beads to pull down EGFP-tagged Split-Miro C-terminus. Immunoprecipitates were blotted and probed with anti-GFP antibody (to detect Split-Miro C-terminus), anti-Miro antibody (to detect Split-Miro N-terminus), and an anti-Milton antibody. Inputs are total lysates (25 μg protein). (E) Cartoon showing Split-Miro and Control constructs with the GFP and Miro antibodies used for immunoprecipitation and western blotting in (D). (F) Representative kymographs of mitochondrial transport in the processes of S2R+ cells transfected with mCherry-tagged Zdk1-MiroC (Control), mCherry-Miro (wt-Miro), and mCherry-Split-Miro (Split-Miro). Scale bars: 2 μm (distance) and 5 seconds (time). G) Distribution of mitochondria run lengths in the processes of S2R+ cells, transfected with control, wt-Miro, and Split-Miro, as shown in F. N = number of mitochondrial runs. One-way ANOVA with Tukey’s post hoc test. (H) Duty cycle analysis describing the average time mitochondria spend moving anterogradely, retrogradely, or p [file pbio.3002273.s004.tif]

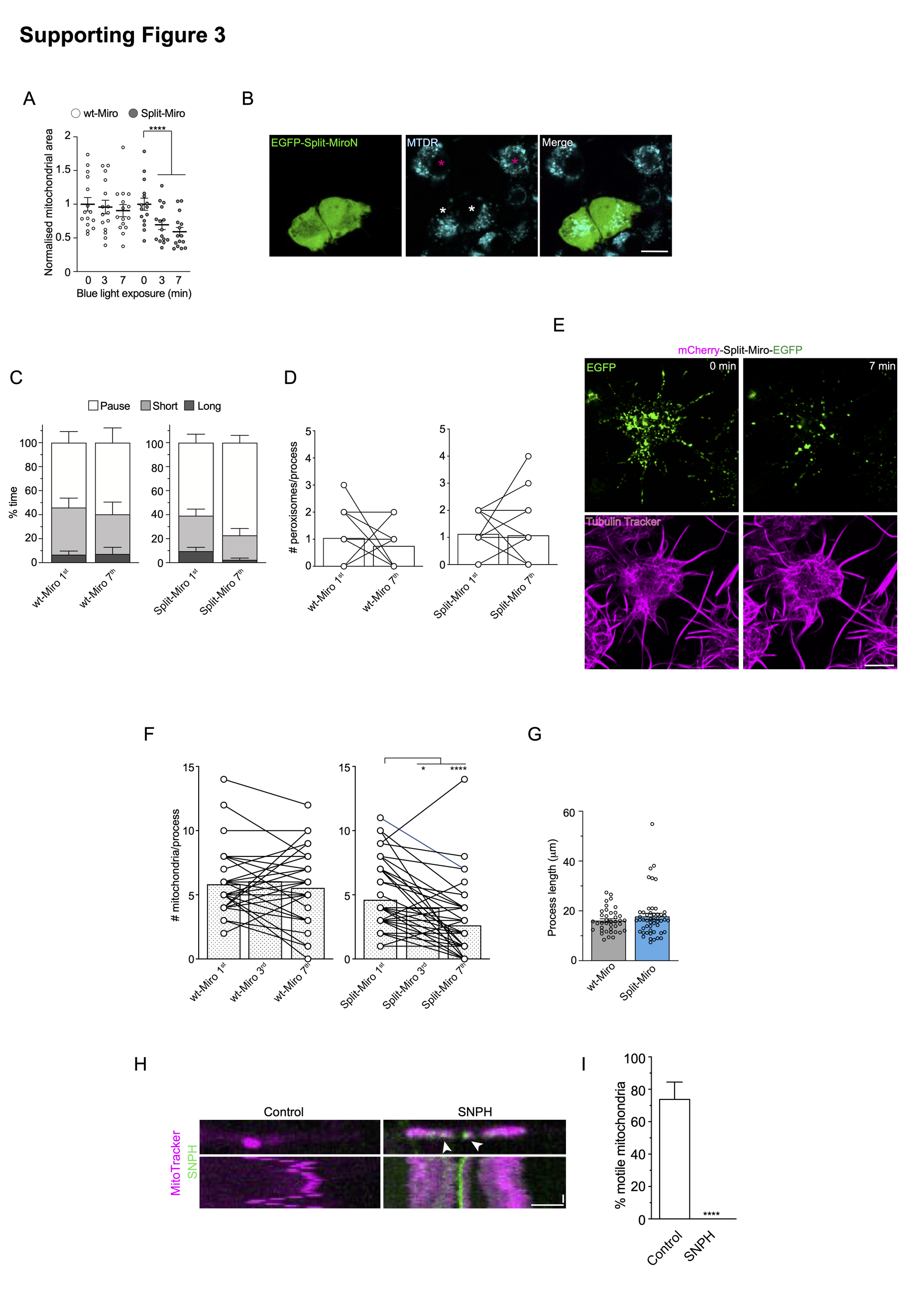

Supplement: S3 Fig — (A) Quantification of the total area covered by the mitochondria within the cell. Each measurement was normalised to the average group value (wt-Miro, Split-Miro) at time point 0. Comparison across time points was performed by repeated measures one-way ANOVA followed by Tukey’s post hoc test. Data are reported as mean ± SEM. Circle, number of cells, from 3 independent experiments. (B) S2 cells are transfected with EGFP-tagged Split-Miro N-terminus (green) and stained with MitoTracker DeepRed (MTDR, cyan). Middle panel: white and magenta stars indicate untransfected and transfected cells, respectively. Scale bar: 10 μm. (C) Duty cycle analysis describes the average time peroxisomes spend on long runs, short runs, or pausing. For each parameter, all peroxisomal values from each cell were averaged and compared between time points. Statistical significance was evaluated by multiple Mann–Whitney tests. (D) Bar chart shows the average peroxisomal content at minute 1 and 7 of time-lapse imaging with blue light. Circles represent the number of peroxisomes within each process. Statistical significance was evaluated by Wilcoxon test. In (C, D), number of processes and cells: wt-Miro = 24, 11, Split-Miro = 39, 16, from 3 independent experiments. There is no significant difference in the motility and number of peroxisomes in each process between timepoints. (E) Representative images showing S2R+ cells transfected with Split-Miro and imaged by time-lapse with blue light for 7 minutes. Exposure to blue light (to induce Split-Miro photocleavage) leads to altered mitochondrial morphology and distribution, without noticeable disorganisation of the microtubule network as detected by the Tubulin Tracker (magenta). Scale bar: 10 μm. Not shown, mCherry-tagged Split-Miro N-terminus. (F) Bar chart shows the average mitochondrial content at minute 1, 3, and 7 of time-lapse imaging with blue light in the processes of S2R+ transfected with either wt-Miro or Split-Miro. Circles represent the [file pbio.3002273.s005.tif]

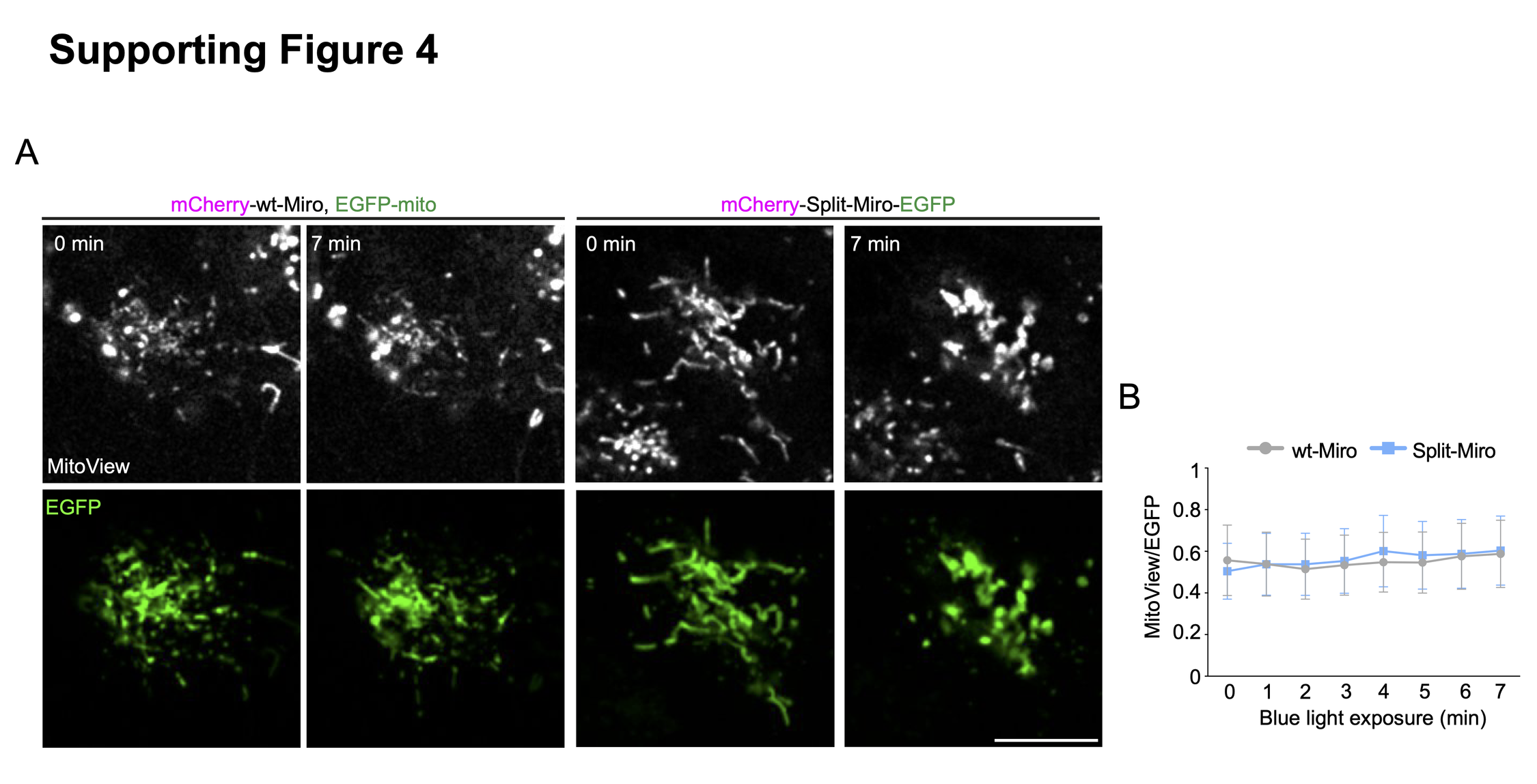

Supplement: S4 Fig — (A) Representative images of cells cotransfected either with wild-type Miro (mCherry-wt-Miro, EGFP-mito) or Split-Miro (mCherry-Split-Miro-EGFP). EGFP signal is used to mark the mitochondria over time; mCherry, not shown. MitoView 405 was used to monitor the mitochondrial membrane potential during the imaging period. Scale bar: 10 μm. (B) Ratio of MitoView and EGFP mitochondrial fluorescence intensity at the time points indicated. Number of cells: wt-Miro = 7, Split-Miro = 7, from 2 independent experiments. Data are shown as mean ± SEM. Each group was analysed by repeated measures one-way ANOVA followed by Dunnett’s post hoc test, with each time point compared to the t = 0 minutes. The data underlying the graphs shown in the figures can be found in S1 Data. (TIF) [file pbio.3002273.s006.tif]

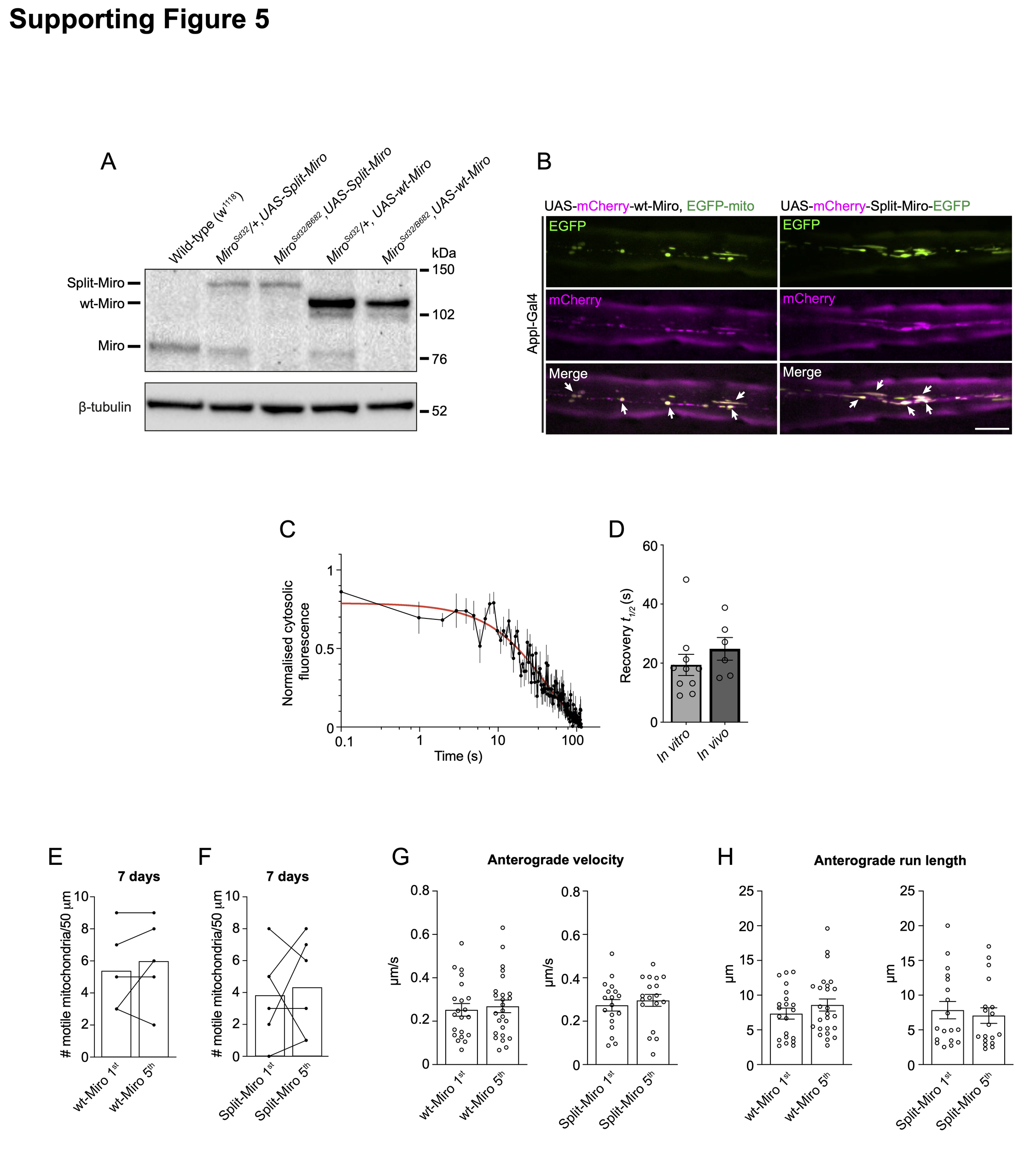

Supplement: S5 Fig — (A) Western blots of lysates from male fly heads of the reported genotypes confirms the expression of UAS-wt-Miro and UAS-Split-Miro, in either Miro heterozygous (miroSd32/+) and null backgrounds (miroSd32/B682), using the Appl-Gal4 driver. UAS-wt-Miro is expressed from attP2; the UAS-MiroN and UAS-MiroC (to reconstitute Split-Miro) were expressed from attP40 and attP2, respectively. The higher molecular weight of wt-Miro and Split-Miro compared to endogenous Miro is consistent with the presence of the mCherry tag (wt-Miro) and mCherry/LOV2 tags (Split-Miro). Please note that the lower expression of Split-Miro compared to wt-Miro does not significantly affect the proportion of rescued flies, as shown in Fig 7A. The top membrane was blotted with an anti-Miro antibody recognising an N-terminal epitope (see also S2E Fig). (B) Representative images of axons in the L3 vein of the adult live fly wing. For colocalisation experiments, flies express mCherry-tagged wt-Miro (attP40) and EGFP-tagged Miro C-terminus (attP2). Split-Miro flies express the N-terminus and C-terminus halves of Split-Miro from attP40 and attP2, respectively. All constructs were expressed under the control of the Appl-Gal4 driver in a miro+/+ background. Arrows highlight examples of colocalised signal. Scale bar: 10 μm. (C) Quantification of the kinetics of Split-Miro recovery after photocleavage in the neurons of the adult fly wing in vivo. UAS-mCherry-Split-MiroN and UAS-MiroC were both expressed from attP40, using the Appl-Gal4 driver. The first time point corresponds to maximum levels of mCherry-Split-MiroN release. Data are shown as mean ± SEM. Red solid line, exponential curve fit (n = 6 wings, from 3 flies). (D) There is no difference in the half-life recovery of Split-Miro after photobleaching in S2R+ cells (in vitro) and in the neurons of the adult fly wing (in vivo). Data are shown as mean ± SEM and were analysed by Mann–Whitney test. Circles, number of cells (in vitro) and wings (in vivo) an [file pbio.3002273.s007.tif]

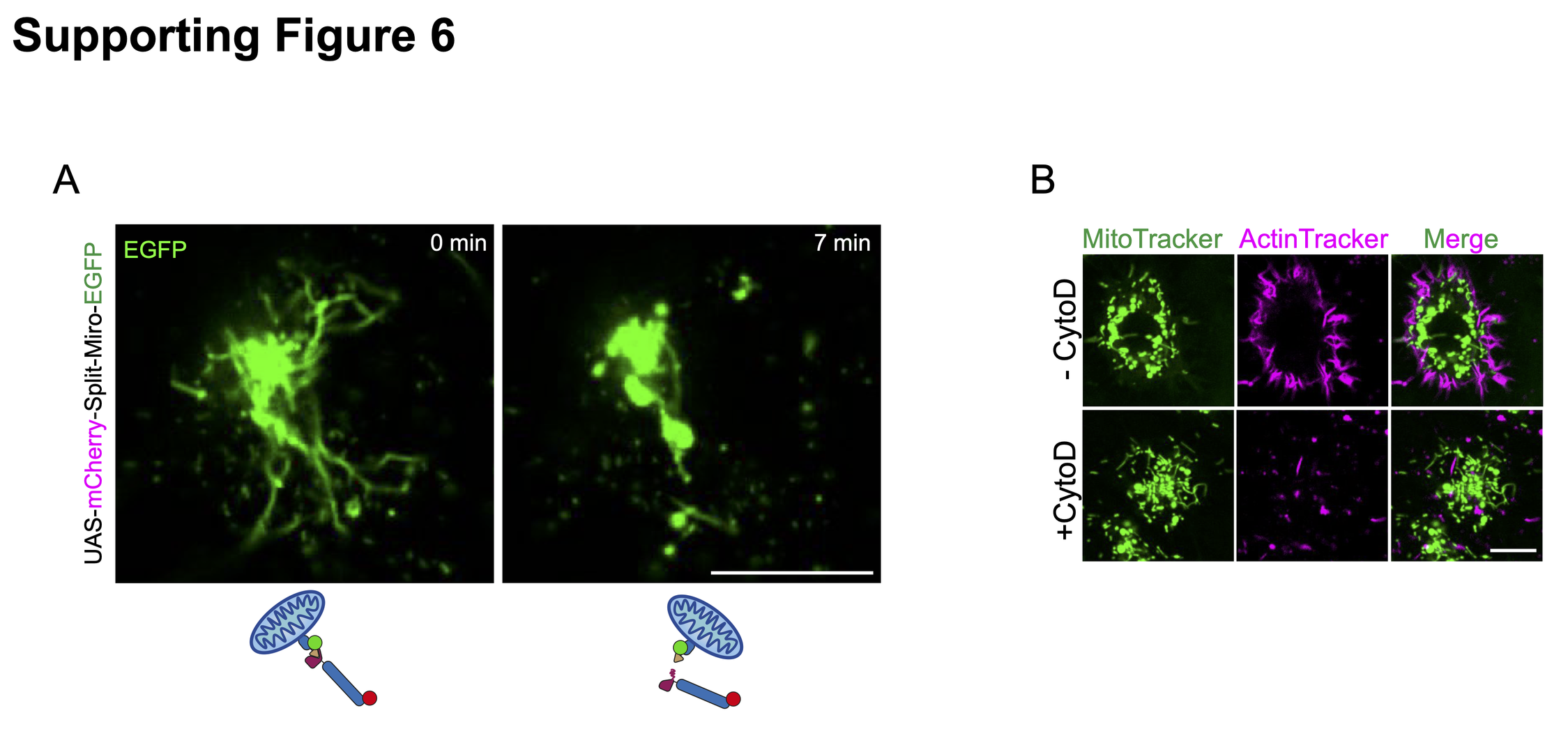

Supplement: S6 Fig — (A) S2R+ cell transfected with mCherry/EGFP-tagged Split-Miro and not treated with cytochalasin D (to maintain an intact actin network) were imaged by time-lapse with a 488-nm laser for 7 minutes. The presence of an intact actin network does not prevent mitochondrial network collapse. Scale bar: 10 μm. Cartoon depicts the reconstitution state of Split-Miro at 0 and 7 minutes (min) under blue light. (B) Representative images of S2R+ cells treated with or without cytochalasin D (cytoD) for 4 hours before imaging. Before imaging, cells were stained with MitoTracker Green (MitoTracker) and ActinTracker DeepRed (ActinTracker) to visualise the mitochondria and the actin network, respectively. Note the depolymerisation of the actin network after cytoD treatment, which is required for process extension in S2R+ cells. Scale bar: 5 μm. (TIF) [file pbio.3002273.s008.tif]
