# Supplementary material for: Optogenetic cleavage of the Miro GTPase reveals the direct consequences of real-time loss of function in Drosophila
Source: PLoS Biol. 2023 Aug 17;21(8):e3002273. doi: 10.1371/journal.pbio.3002273 (PMC10465005; doi:10.1371/journal.pbio.3002273)
Supplement: S1 Table — (DOCX) [file pbio.3002273.s009.docx]

**Supporting Table 1.** Plasmids used in this study.

| **Construct number** | **Insert** | **Backbone** | **Cloning method, construct assembly and DNA sources** |
| --- | --- | --- | --- |
| #1. | (Empty vector) | pAc5.1/V5-HisB (pAc5.1) | Gift of Alex Whitworth (MRC-MBU, Cambridge, UK). |
| #2. | (Empty vector) | pUASTattB | Gift of Manolis Fanto (King’s College London, London, UK). |
| #3 | SPLICS-L | pT2-DsRed-UAS | Gift of Tito Calì (University of Padova, Padova, Italy). |
| #4 | ^KpnI^mCherry-Miro^XbaI^ | pAc5.1/V5-HisB | NEBuilder HiFi DNA Assembly using primers #13,#14 (for mCherry) and #8,#2 (for Miro).  mCherry was PCR amplified from Addgene #81041 and fused to the *Drosophila* Miro-RE/RF cDNA sequence. |
| #5 |  | pUASTattB | Restriction enzymes-based cloning. Insert cut and pasted from construct #4. |
| #6 | ^KpnI^EGFP-MiroN-LOV2 T406-407A^NotI^ | pAc5.1/V5-HisB | NEBuilder HiFi DNA Assembly using primers #6,#7 (for EGFP); #8,#9 (for MiroN) and #10,#11 (for LOV2 T406-407A).  EGFP was PCR amplified from Clontech pEGFP-N1. MiroN, encompassing the aa 1-642 of *Drosophila* Miro, was PCR amplified from endogenous Miro-RE/RF. LOV2 T406-407A was obtained by site-directed mutagenesis of mCherry-MiroN-LOV2 wild type (wt). |
| #7 | ^KpnI^mCherry-MiroN-LOV2 T406-407A^NotI^ | pAc5.1/V5-HisB | NEBuilder HiFi DNA Assembly using primers #13,#14 (for mCherry) and #8,#11 (for MiroN-LOV2 T406-407A).  mCherry was PCR amplified from Addgene #81057. MiroN-LOV2 T406-407A was amplified from EGFP-MiroN-LOV2 T406-407A (construct #6). |
| #8 |  | pUASTattB | Restriction enzymes-based cloning. Insert cut and pasted from construct #7. |
| #9 | mCherry-MiroN-LOV2 wt | pAc5.1/V5-HisB | The construct was obtained by site-directed mutagenesis of construct #7 using primers #23, #24. |
| #10 | ^NotI^mCherry-Zdk1-MiroC^XbaI^ | pAc5.1/V5-HisB | NEBuilder HiFi DNA Assembly using primers #3,#4 (for mCherry-Zdk1) and #5,#2 (for MiroC).  mCherry-Zdk1 was PCR amplified from Addgene #81057 and fused to MiroC (encompassing the aa 643-674 of *Drosophila* Miro). |
| #11 | ^KpnI^EGFP-Zdk1-MiroC^XbaI^ | pAc5.1/V5-HisB | NEBuilder HiFi DNA Assembly using primers #6,#15 (for EGFP) and #16,#2 (for Zdk1-MiroC).  EGFP was PCR amplified from Clontech pEGFP-N1 and fused to Zdk1-MiroC amplified from construct #10. |
| #12 |  | pUASTattB | Restriction enzymes-based cloning. Insert cut and pasted from construct #11. |
| #13 | ^NotI^Zdk1-MiroC^XbaI^ | pAc5.1/V5-HisB | NEBuilder HiFi DNA Assembly using primers #12,#2.  Zdk1-MiroC was amplified from construct #10. |
| #14 |  | pUASTattB | Restriction enzymes-based cloning. Insert cut and pasted from construct #13. |
| #15 | GFP-SKL | pGG101 | AcGFP-SKL (gift of Gohta Goshima, Nagoya University, Nagoya, Japan) |
| #16 | ^KpnI^Mito4xGCaMP6f^NotI^ | pAc5.1/V5-HisB | NEBuilder HiFi DNA Assembly using primers #17,#18.  Insert PCR-amplified from Addgene #127870. |
| #17 | ^KpnI^EGFP^EcoRI^SNPH^XbaI^ | pAc5.1/V5-HisB | Restriction enzymes-based cloning. Human SNPH (Gift of Zu-Hang Sheng, NIH, USA) was cut and pasted into an EGFP-pAc5.1/V5-HisB vector. |
| #18 | ^KpnI^EBFP^EcoRI^SNPH^XbaI^ | pAc5.1/V5-HisB | NEBuilder HiFi DNA Assembly using primers #19,#20 for EBFP.  EBFP was PCR amplified from Clontech pEBFP-C1 (gift of Marc-David Ruepp, King’s College London, London, UK) and fused to human SNPH (Gift of Zu-Hang Sheng, NIH, Bethesda, USA). |
| #19 | ^BamHI^mito-ER.SPLICS^XbaI^ | pAc5.1/V5-HisB | Restriction enzymes-based cloning. Mito-ER.SPLICS was cut and pasted from construct #3 into the pAc5.1/V5-HisB vector. |
