# Supplementary material for: Optogenetic cleavage of the Miro GTPase reveals the direct consequences of real-time loss of function in Drosophila
Source: PLoS Biol. 2023 Aug 17;21(8):e3002273. doi: 10.1371/journal.pbio.3002273 (PMC10465005; doi:10.1371/journal.pbio.3002273)
Supplement: S2 Table — For NEBuilder HiFi DNA Assembly, bold fonts indicate primer overlap with the amplified gene product, underline indicated overlap with the adjacent insert or plasmid sequence. For site-directed mutagenesis, bold fonts indicate mutated nucleotides. For dsRNA template production, underline indicates T7 promoter sequence which precedes a sequence overlapping with either Miro 3′-UTR (dsRNA) or a nontargeting sequence within the pT2-DsRed-UAS plasmid backbone (Control). (DOCX) [file pbio.3002273.s010.docx]

**Supporting Table 2.**

| **Primer number** | **Primer name** | **Direction** | **Sequence (5’-3’)** |
| --- | --- | --- | --- |
| NEBuilder HiFi DNA Assembly | | | |
| #1. | Miro (overlap to pAc5.1) | Fwd | **TCCAGAGACCCCGGATCGGG**GTACCATGGGACAGTACACGGCGTCGCAGCGCAAG |
| #2. | Miro (overlap to pAc5.1) | Rvs | **ACCTTCGAACCGCGGGCCCT**CTAGACTAACGGGTGTGGGCTCCGGCAGCACTTAT |
| #3. | mCherry (overlap to pAc5.1) | Fwd | **CAGATATCCAGCACAGTGGC**GGCCGCatggtgagcaagggcgaggaggataacatg |
| #4. | Zdk1 (overlap to Miro-Cterm) | Rvs | **AGTCCCGCCTTTAACCACAG**gctaccaccagaaccaccttttggggcctg |
| #5. | Miro-Cterm (overlap to Zdk1) | Fwd | **aaggtggttctggtggtagc**CTGTGGTTAAAGGCGGGACTAGGAGTGGCC |
| #6. | EGFP (overlap to pAc5.1) | Fwd | **TCCAGAGACCCCGGATCGGG**GTACCatggtgagcaagggcgaggagctgttcacc |
| #7. | EGFP (overlap to Miro-Nterm) | Rvs | **GACGCCGTGTACTGTCCCAT**cttgtacagctcgtccatgccgagagtgat |
| #8. | MiroNterm (overlap to EGFP) | Fwd | **gcatggacgagctgtacaag**ATGGGACAGTACACGGCGTCGCAGCGCAAG |
| #9. | MiroNterm (overlap to LOV2) | Rvs | **gcagccaaggatccagaacc**CTTGGGGTCCTCCGTCATCAGGCCGAATTG |
| #10. | LOV2 (overlap to Miro-Nterm) | Fwd | **TGATGACGGAGGACCCCAAG**ggttctggatccttggctgctgcacttgaa |
| #11. | LOV2 (overlap to pAc5.1) | Rvs | **CGGGCCCTCTAGACTCGAGC**GGCCGCttaaagttcttttgccgcctcatcaatatt |
| #12. | Zdk (overlap to pAc5.1) | Fwd | **CAGATATCCAGCACAGTGGC**GGCCGCATGggttctggatccatggtggataacaaattc |
| #13. | mCherry_2 (overlap to pAc5.1) | Fwd | **TCCAGAGACCCCGGATCGGG**GTACCatggtgagcaagggcgaggaggataacatg |
| #14. | mCherry (overlap to Miro-Nterm) | Rvs | **GACGCCGTGTACTGTCCCAT**cttgtacagctcgtccatgccgccggtgga |
| #15. | EGFP (overlap to Zdk1) | Rvs | **tccaccatggatccagaacc**cttgtacagctcgtccatgccgagagtgat |
| #16. | Zdk1 (overlap to EGFP) | Fwd | **Gcatggacgagctgtacaag**ggttctggatccatggtggataacaaattc |
| #17. | Mito4xGCaMP6f (overlap to pAc5.1) | Fwd | **TCCAGAGACCCCGGATCGGG**GTACCATGAGCGTGCTGACACCTCTGCTGC |
| #18. | Mito4xGCaMP6f (overlap to pAc5.1) | Rvs | **CGGGCCCTCTAGACTCGAGC**GGCCGCTCACTTGGCGGTCATCATCTGGACAAACTC |
| #19. | EBFP (overlap to pAc5.1) | Fwd | **TCCAGAGACCCCGGATCGGG**GTACCATGGTGAGCAAGGGCGAGGAGCTGTTC |
| #20. | EBFP (overlap to SNPH) | Rvs | **GGCCGCTGCCCGGCATGGTG**AATTCCTTGTACAGCTCGTCCATGCCGAGAGTG |
| Site-directed mutagenesis | | | |
| #21. | LOV2 mut | Fwd | ATCCTTGGCT**G**CT**G**CACTTGAACGTA |
| #22. | LOV2 mut | Rvs | CCAGAACCCTTGTACAGC |
| #23. | LOV2 wt | Fwd | ATCCTTGGCT**A**CT**A**CACTTGAACGTA |
| #24. | LOV2 wt | Rvs | CCAGAACCCTTGGGGTCC |
| dsRNA template production | | | |
| #25. | Miro dsRNA | Fwd | TAATACGACTCACTATAGGgagGGGAATTCACTAGGATAAGGGGA |
| #26. | Miro dsRNA | Rvs | ATTATGCTGAGTGATATCCctcGCCATTAAATATCACTATATGTTAATCCA |
| #27. | Control dsRNA | Fwd | TAATACGACTCACTATAGGgag |
| #28. | Control dsRNA | Rvs | ATTATGCTGAGTGATATCCctcctgtagcccaagttgttgatattat |
