# Supplementary material for: Optogenetic cleavage of the Miro GTPase reveals the direct consequences of real-time loss of function in Drosophila
Source: PLoS Biol. 2023 Aug 17;21(8):e3002273. doi: 10.1371/journal.pbio.3002273 (PMC10465005; doi:10.1371/journal.pbio.3002273)
Supplement: S3 Table — (DOCX) [file pbio.3002273.s011.docx]

**Supporting Table 3.**

| **Strain** | **Source** |
| --- | --- |
| Oregon-R | Bloomington Drosophila Stock Center (BDSC), #5 |
| w^1118^ | BDSC, #6326 |
| UAS-mito::GFP | BDSC, #8442 |
| Appl-Gal4 | BDSC, #32040 |
| miro^B682^/TM6c | BDSC, #52003 |
| miro^Sd32^/T(2;3),CyO:TM6b | BDSC, #52002 |
| 20xUAS-GCaMP5G | BDSC, #56500 |
| w; 5xUAS-mCherry::MiroN-LOV2 T406-407A (attP40)/CyO | This study |
| w;; 5xUAS-EGFP::Zdk1-MiroC (attP2)/TM6c | This study |
| w;; 5xUAS-Zdk1-MiroC (attP2)/TM6c | This study |
| w; 5xUAS-Zdk1-MiroC (attP40)/CyO | This study |
| w; 5xUAS-mCherry::Miro wt (attP40)/CyO | This study |
| w;; 5xUAS-mCherry::Miro wt (attP2)/TM6c | This study |
| Genotypes for behavioural assay | |
| w, Appl-Gal4/Y; 5xUAS-mCherry::MiroN-LOV2 T406-407A (attP40)/+; 5xUAS-Zdk1-MiroC (attP2), miro^Sd32^/miro^B682^ | |
| w, Appl-Gal4/Y; +/+; 5xUAS-mCherry::Miro wt (attP2), miro^Sd32^/miro^B682^ | |
| w, Appl-Gal4/Y; 5xUAS-mCherry::MiroN-LOV2 T406-407A (attP40)/+; 5xUAS-Zdk1-MiroC (attP2), miro^Sd32^/EGFP-SNPH (attP2), miro^B682^ | |
| Genotypes for live imaging of mitochondrial transport | |
| w, Appl-Gal4/+; UAS-mito::GFP/+; 5xUAS-mCherry::Miro wt (attP2), miro^Sd32^/+ | |
| w, Appl-Gal4/+; 5xUAS-mCherry::MiroN-LOV2 T406-407A (attP40)/ +; 5xUAS-EGFP-Zdk1-MiroC (attP2), miro^Sd32^/+ | |
| Genotypes for live imaging of neuronal activity | |
| w, Appl-Gal4/+; 5xUAS-mCherry::Miro wt (attP40)/+; 5xUAS-Zdk1-MiroC (attP2), miro^Sd32^/20xUAS-GCaMP5G | |
| w, Appl-Gal4/+; 5xUAS-mCherry::MiroN-LOV2 T406-407A (attP40)/+; 5xUAS-Zdk1-MiroC (attP2), miro^Sd32^/20xUAS-GCaMP5G | |
