# Supplementary material for: Optogenetic cleavage of the Miro GTPase reveals the direct consequences of real-time loss of function in Drosophila
Source: PLoS Biol. 2023 Aug 17;21(8):e3002273. doi: 10.1371/journal.pbio.3002273 (PMC10465005; doi:10.1371/journal.pbio.3002273)
Supplement: Attachment — Submitted filename: Mattedi et al_Rebuttal.pdf [file pbio.3002273.s018.pdf]

We are grateful to the reviewers for their thorough evaluation of our work. We have performed a series of new experiments and analyses in response to their comments. We have also rewritten several sections of the manuscript to clarify the issues that were raised. Thus, our revised manuscript addresses all issues raised by the reviewers. In the following we provide a point-by-point reply that highlights additions, amendments, and changes in response to the reviewer comments.
Throughout the rebuttal, the 'line' numbers refer to the clean version of the uploaded manuscript without track changes.

Rev. 1:
This manuscript by Mattedi et al. describes the use of optogenetic manipulation of the Miro GTPase in Drosophila. This is a challenging but potentially very useful approach to study the acute effects of loss of a functional Miro in an animal model. Most of the studies were conducted in insect cells but the study included an effort also to analyze the activity of adult flies. I find the manuscript very interesting, the experiments have been carried out in an appropriate manner and the text is well written and follows a logical flow. I only have some minor comments:

1. The design of the split-Miro construct result in a loss of mitochondrial targeting of Miro since the LOV2:Zdk1 pair is inserted between the C-terminal GTPase domain and the transmembrane domain of Miro. Blue light releases the major part of Miro from the transmembrane domain; however, I believe the GTPase domain and EF hands are still intact and active. If I understand correctly, the result is therefore not really a loss of function but rather a loss of mitochondrial targeting. It is clear that the mislocalization negatively affects the mitochondrial motility but could there be any adverse effects by the released Miro deletion mutant? The released Miro variant could maybe sequester Miro binding proteins in the cytoplasm and the effects might not be apparent on the time-scale examined in the experimental design. What is the view of the authors on this possibility?

We agree with the reviewer it is conceivable that Miro domains could still be intact and functional upon release from the mitochondrial membrane. As such, we cannot rule out these domains might result in a gain of cytoplasmic function which could have secondary effects in a longer timescale. The same logic would apply to potential sequestration of binding partners away from the mitochondria and into the cytoplasm. The rapid timescale at which we observe the cellular and behavioural phenotypes, however, are unlikely to be explained by this scenario. Instead, we think that rapid re-localisation of most of the protein into the cytoplasm leads to a loss of canonical Miro functions carried out at the mitochondrial outer membrane, which is responsible for the observed phenotypes.
During our study, we also noticed that overexpressing the Miro N-terminal moiety alone, which remains distributed throughout the cytoplasm, does not cause any gross mitochondrial morphological aberration or network collapse and we added an example of this control experiment (S3B Fig). This agrees with previous studies in which transient overexpression of mammalian Miro1/2 lacking the transmembrane domain (similar to our Split-MiroN construct) did not alter mitochondrial distribution or cause any detectable mitochondrial morphological phenotype (for example, Fransson et al. 2006 PMID: 16630562; Glater et al. 2006 PMID: 16717129). We have now modified the main text and added these references to clarify this aspect (lines 247-251).

2. I am a bit surprised by the increased activity of the adult flies described in Fig.5. It is not entirely clear what is meant by "activity" for a non-fly expert. What precisely is measured? I thought that a decreased mitochondrial motility would result in decreased neuro-muscular function and hence reduced activity. Why do we see the opposite in this experimental set up? The authors could elaborate a bit more on this finding.

In Fig. 5 (now Fig. 7) we measure fly locomotor activity. Graphs 7C, F, I (% activity) show the percentage of time the flies spend moving with animals defined as 'active' if displaying bouts of activity of at least 2 mm/s. We detail this in the 'behavioural assay' section of the methods.
In our manuscript we acknowledge the evidence that both increasing and reducing Miro abundance in the *Drosophila* nervous system is sufficient to rescue fly climbing activity in models of neurodegeneration (Panchal et al., 2018, PMID: 32747449; Shaltouki at al., 2020, PMID: 29923074) (lines 437-438). The mechanisms underlying this contrasting effect are not clear although it is possible that the tissue specificity of these manipulations might underpin the different phenotypes. We have expressed Split-Miro in the nervous system using a pan-neuronal driver which might account for compensatory effects at the systems level. It would have been interesting to see if manipulating the mitochondria through Split-Miro in specific neuronal subtypes or in non-neuronal tissues may lead to different behavioural outputs. Currently, however, testing this hypothesis is limited by the requirement of pan-neuronal expression to rescue the lethality associated with a *miro*$^{-/-}$ background. As such, our locomotor assay is a readout of overall nervous system performance.

In our original discussion, we mention that "*[…] reducing mitochondrial number positively correlates with activity-dependent vesicular release at the presynapses […] (10,58) and with miniature excitatory junction potentials at the Drosophila NMJ (4)*". We also speculate that "*It is conceivable that Split-Miro triggers an acute imbalance of neuronal homeostasis and synaptic transmission, which leads to augmented locomotor activity*". We have now expanded this point by producing new genetic backgrounds to perform a new set of experiments in which we record spontaneous activity of live neurons using the GCaMP5 calcium indicator. We found that bouts of neuronal activity are more frequent in Split-Miro flies compared to wt-Miro flies exposed to 488-nm light, while the intensity of the response is reduced (Fig. 6). This suggests that, although the basal activity of the neurons is dampened by Split-Miro, more neurons are responsive. This observation is consistent with a distributed network response described for neural ensembles and manifolds (Aery Jones and Giocomo, 2023, PMID: 36542882; Langdon et al 2023, PMID: 37055616) in which an enlarged population of neurons is recruited to maintain network performance despite dampened activity of individual cells. Overall, these data support the notion that Split-Miro may affect fly behaviour by triggering an imbalance of neuronal activity. We are mindful that we performed these experiments in the wing neurons and, although these cells can relay signals to affect motor phenotypes (see for example, Raad et al., 2016, PMID: 27160896; Zhang et al., 2022, PMID: 35243214), we are cautious not to generalise our findings to all neurons in the fly (lines 598-600).

3. Please remove the sentence "To our knowledge, this is the first example of tunable animal behavior by real-time loss of protein function" (lines 68-69). This statement is irrelevant.

We have removed this statement as requested by the reviewer. At the same time, we feel it is justified to highlight that our data provides a proof of concept that animal behaviour can be tuned by real-time loss of protein function, which has not been reported before. Thus, we have now added *"we provide proof of concept"* to the previous sentence (lines 71-72) to convey this message, which we think is in line with the 'priority claims' guidelines from PLOS Biology (https://journals.plos.org/plosbiology/s/submission-guidelines#loc-introduction). We hope the reviewer finds this choice of language acceptable.

Rev. 2:
The study by Mattedi et al developed an optogenetic 'Split-Miro' system controlling Miro-dependent mitochondrial functions in Drosophila that is based on the light-oxygen-voltage-

sensing domain 2 (LOV2) and its binding partner ZDark1 (zdk1). The feasibility of the Split-Miro system is demonstrated in Drosophila S2R+ cells. Acute dissociation of Split-Miro in S2R+ cells disrupts the processivity of microtubules-based mitochondrial transport and causes a rearrangement of the mitochondrial network, as it has been previously observed for loss of function mutations. However, dissociation of Split-Miro had no effect on the ability of mitochondria to buffer calcium in contrast to previous studies. The study further shows the Split-Miro protein is at least partially functional in intact animals that acute inactivation of Miro function causes an unexplained hyperactivity in flies.

1) The experiment showing the dissociation and reassociation of Split-Miro is not convincing (Fig 1D-F). The representative image shows a diming of mitochondria-associated mCherry-Split-MiroN but not the expected increase of the mCherry signal in the cytosol. I am also confused why only MiroN but not MiroC is tagged in this experiment.

Since it is critical to demonstrate how well the Split-Miro system dissociates in response to blue light and reassociates in its absence, I suggest modifying the experiment by using a tagged (instead of untagged) Split-MiroC version. This will allow a quantification of a) how much mCherry-Split-MiroN becomes cytosolic and b) how much mCherry-Split-MiroN remains associated with mitochondria in the presence of blue light. The same principle should be applied to reassociation.

We apologise for the lack of clarity regarding the tagging strategy of Split-Miro. Our aim was to show the versatility, along with the efficiency, of the Split-Miro system by using different combinations of tags. To clarify our approach, we have simplified and modified the previous Fig. 1D by adding the MitoTracker staining to show the extent of release and reassociation of mCherry-Split-MiroN. We also changed the colour-code of the image so that the re-distribution of mCherry-MiroN can be better appreciated. This is now Fig. S1B-E.

At the same time, we recognise that the efficiency of dissociation and reconstitution of double-tagged Split-Miro might be different than if tagged only with one fluorophore. As suggested by the reviewer, we therefore have now expanded this part by also performing a new set of experiments in which we quantify the release and reassociation of the mCherry-Split-MiroN using a double-tagged Split-Miro (i.e. mCherry-Split-Miro-EGFP), including data showing the relative proportion of mCherry-Split-MiroN becoming cytosolic and remaining associated with the mitochondria after photocleavage, and the proportion of mCherry-Split-MiroN reassociating at the mitochondria to reconstitute Split-Miro. This is now detailed in Fig. 1C-G. We found the kinetics of dissociation and reconstitution of mCherry-Split-Miro-EGFP to be very similar to the kinetics of mCherry-Split-Miro and we observe near-complete Split-Miro dissociation and reconstitution at the mitochondria. We thank the reviewer for the opportunity to clarify this aspect of our Split-Miro design.

2) Fig 1C. The shown reconstitution of Split-Miro at mitochondria in the absence of blue light requires quantification to clearly demonstrate the efficiency of the Split-Miro system.

Our aim was to show the versatility of the Split-Miro system by using different combinations of tags. With this combination (EGFP-Split-Miro-mCherry), however, accurate quantification of the kinetics of dissociation/reassociation of the EGFP-tagged Split-MiroN is impeded by the fact that Split-Miro is cleaved by imaging the EGFP with blue light, rendering the quantification of the reassociation kinetics not possible. We would not expect a striking difference to using the inverted mCherry-Split-Miro-EGFP combination (Fig. 1B-G), as the structures of two tags are very similar. Therefore, here we have only shown the co-localisation of the N-ter and C-ter of Split-Miro at the mitochondria as an indication of Split-Miro reconstitution. We think this figure is

valuable if others wish to use this tag combination in future studies. However, because of the new data in Fig. 1, we have moved this panel to the supplementary material (now S1A Fig).

3) The age-dependent effects of acute Split-Miro inactivation increasing locomotor activity are interesting but unexplained. Any follow-up experiment providing some evidence to explain this effect will significantly strengthen the manuscript.

We agree with the reviewer that it would be interesting to provide a more in-depth explanation of why age exacerbates the effect on locomotor activity, which is already detectable in 2-day-old flies (now Fig. 7), following acute Split-Miro inactivation. We have performed a new set of experiments by generating new genetic backgrounds and show that Split-Miro impacts on neuronal activity in 2-day-old flies (now Fig. 6). On one hand, these data are consistent with previous studies showing that "*[…] reducing mitochondrial number positively correlates with activity-dependent vesicular release at the presynapses […]*". On the other hand, the data support the notion that Split-Miro could affect fly behaviour by triggering an imbalance in neuronal activity, as speculated in our original discussion: '*It is conceivable that Split-Miro triggers an acute imbalance of neuronal homeostasis and synaptic transmission, which leads to augmented locomotor activity*'. We have also attempted to measure neuronal activity in older flies. Unfortunately, obtaining reliable *in vivo* measurements was often not possible in this case due to a mix of increased GCaMP fluorescence in the axonal bundle and dimmer overall signal that amplified the noise and masked bouts of neuronal activity. We include a still image below (Rebuttal Fig. 1), to show the contrast to the clearer and quantifiable images obtained in younger flies (Fig. 6). We have now expanded the last two paragraphs of our discussion and speculate that 'in older flies, a potentially compromised cellular state might contribute to amplify this response' (lines 595-596).

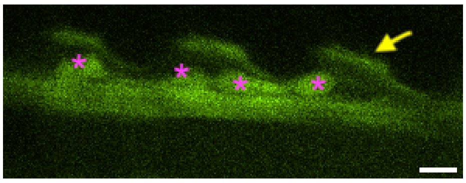

Rebuttal Fig. 1. GCaMP5 signal in the wing marginal neurons of 8-day old flies. Please note the high background signal from the wing cuticle (yellow arrow). Higher background means that bouts of neuronal activity are often masked and the soma of the neurons (magenta stars) are not always clearly distinguishable from the axonal bundle.

In addition to the experiment in which we measure neuronal activity, we have also generated new genetic backgrounds to perform a new set of experiments to analyse mitochondrial trafficking *in vivo* in young and aged flies (Fig. 5). We provide evidence that Split-Miro photocleavage acutely reduces mitochondrial trafficking *in vivo* in the wing neurons of 2-day-old flies, although this is not the case in older flies (Fig. 5 and S5E-H Fig), when transport is known to decline in this system (Vagnoni et al., 2016, PMID: 26598558; Vagnoni and Bullock, 2018, PMID: 29606421). These data suggest that although the mild effect on fly activity observed at 2 days (Fig. 7) correlates with Split-Miro-dependent reduction in mitochondrial trafficking (and imbalanced neuronal activity), the increase in locomotor activity in aged flies cannot be explained by reduced trafficking. In our extended discussion, we now argue that *"[…] the remarkable rescue of Split-Miro-induced hyperactivity in older animals by SNPH overexpression could conceivably occur via further reduction of the remaining mitochondrial transport in older neurons or via a direct effect on synaptic mitochondria (40). Overall, our data point to an important role of neuronal mitochondrial mobility for animal behaviour […]"*.

We feel that our amended discussion provides a reasonable rationalisation of our data. We recognise, however, that the mechanism underlying a more pronounced hyperactivity during ageing is likely more complex, and so we have modified the title of the relevant paragraph to "*Inducible hyperactivity of Split-Miro flies is exacerbated by age"* (line 432) to factually summarise the data, rather than implying the effect we observe is strictly age-dependent. Also note that in the discussion we state that "*hyperactivity becomes more pronounced with age*" (line 590) rather than stating that it is age-dependent (i.e., only seen in older flies).
We wish to emphasise that, although we would really like to better characterise the mechanisms underlying enhanced hyperactivity especially during ageing, this is not the mainstay of our current work. We agree, however, this is an important and timely question that deserves further investigation and we have expanded the last paragraph of the discussion to make this clear, integrating our new data while acknowledging that the exacerbated hyperactivity is unexplained (lines 592-593). We are also mindful that we performed these experiments in the wing neurons and, although these cells can relay signals to affect motor phenotypes (see for example, Raad et al., 2016, PMID: 27160896; Zhang et al., 2022, PMID: 35243214), we are cautious not to generalise our findings to all neurons in the fly (lines 598-600).
Partly due to the complexity of undertaking such studies in adult animals of complex genetic backgrounds and different ages, we are now actively pursuing this aspect in a separate project that significantly diverges from the focus of the current study.

4) The suggested model for Miro-mediated regulation of mitochondrial motility is not new and should be eliminated from the manuscript. Previously, Glater et al (2006, JCB) showed that a truncated Milton protein containing residues 847-1,116 can still associate with mitochondria even though Miro binding is abolished. Therefore, Glater et al hypothesized Milton-motor complexes can also bind to an unidentified mitochondrial protein in a Miro-independent manner.

We agree with the reviewer that Glater et al. (2006, JCB; PMID: 16717129) hypothesise that the Milton-motor complexes can bind to the mitochondria in a Miro-independent manner in COS7 cells. This was later further confirmed by Lopez-Domenec et al. (2018, EMBO J; PMID: 29311115) showing Miro-independence for mitochondrial redistribution mediated by Trak-kinesin complexes in mouse embryonic fibroblasts. We cite both studies in our manuscript, along with more recent *in vitro* reconstitution studies in which Miro was shown to directly bind the motor complex thus also suggesting 'Trak/Milton-independence' for mitochondrial motility. We now add these references also to the original Fig. 6 legend (now Fig. 2I): *"This model is consistent with previous reports describing Miro-independent mitochondrial motility (Glater 2006, Lopez-Domenec 2018)"* (lines 209-210).
We wish to emphasise that with our speculative model we want to facilitate the interpretation of our mitochondrial trafficking data in S2R+ cells in which we show both an essential role for Miro in the activation of bidirectional mitochondrial motility (via Split-Miro overexpression) as well as Miro-independence with regards to motile properties such as velocity and run length (via Split-Miro photocleavage) within the same experimental series. We believe this finding is specifically attainable by using our experimental setup with Split-Miro which we regard as being an important part of our manuscript.
Our results led us to hypothesise that the motor complexes, once recruited onto the mitochondria in a Miro-dependent manner, are stabilised on at least a proportion of the mitochondria in a Miro-independent way, and we recognise this might or might not occur with the involvement of Milton [(line 202-203: "*Split-Miro links directly or indirectly (for example, via Milton/Trak, not depicted) to the motor proteins*"].
Thus, we believe our model is different from the one put forward by Glater *et al* and so not irrelevant, rather it aids towards the interpretation of our findings in a measured

way while integrating our data with the existing literature. Because only part of our manuscript focuses on mitochondrial trafficking, we have decided to add our cartoon as an additional panel to Fig. 2 to aid the interpretation of this specific dataset.
We hope the reviewer will appreciate our effort to integrate our new findings with the knowledge deriving from previous important studies.

5) In the discussion section the authors hypothesize that the impairment of mitochondrial Ca2+ homeostasis that has been demonstrated with Miro loss of function approaches may be a secondary effect of Miro loss of function. While this is reasonable, the authors should also mention the possibility that this presumed and well documented function of Miro could be linked to the TM domain or the few amino acids of the C-terminus of Miro, which are known to interact with Sam and MICOs complexes. Accordingly, the remaining Split-MiroC may still mediate mitochondrial Ca2+ homeostasis in the presence of blue light.

We agree with the reviewer that we cannot assume that the short mitochondrial targeting sequence is inert at the mitochondrial membrane purely based on current lack of evidence for a functional role. We added this possibility in the discussion of our manuscript (lines 560-563): "*Because Miro was shown to interact with MCU and the Sam/MICOS complexes (Niescier 2018, Modi 2019), presumably via direct interaction with the Miro transmembrane domain, we cannot exclude that the short mitochondrial targeting sequence might still mediate* $[Ca^{2+}]_m$ *homeostasis*".

Minor
Fig 1a: The Figure is drawn as if a lov2-ZDK1 cassette is being inserted into Miro, which is confusing. I suggest showing the two generated constructs (MiroN and -C) independently.

We have amended the figure and the figure legend as suggested.

Rev. 3:
This is a potentially interesting manuscript that focuses on the dynamic role of Miro GTPases for regulating mitochondrial trafficking. Miro proteins have long been know to play important roles in regulating mitochondrial distribution and morphology by coupling mitochondria to microtubule dependent transport. Most studies to date have used either loss or gain of function approaches in a wide variety of in vitro and in vivo model systems including worms, flies, mice and human cells. However the implications of acute disruption of Miro on mitochondrial dynamics are less well understood. To address this the authors have used an optogenetic technique allowing for reversible light based disruption of drosophila Miro function by taking advantage of the LOV2 system whereby a LOV2 domain is fused to the C-terminus of the (N-terminal) cytoplasmic part of dMiro while the Miro TM has an N-terminal ZDK1 domain. In the dark state these two domains reconstitute a protein complex (apparently making a functional Miro) that can then be disrupted by green light and hence allowing for rapid optogenetic Miro 'photocleavage'. On the whole the experiments are well performed throughout.

While the approach is elegant and has elements of state of the art and there are a number of potentially interesting findings, enthusiasm is dampened by the lack of major new mechanistic insight regarding Miro function gleaned from the approach. The authors do not seem to have gone into much depth with their model leaving many issues unaddressed despite the potential of the system. The contribution to advancing understanding Miro function therefore remains quite superficial.

We thank the reviewer for their appreciative comments regarding our experimental approach and its versatility for *in vitro* and *in vivo* studies for *Drosophila* use. We politely disagree with the assessment that we only superficially contribute to the understanding of Miro function. We have purposedly chosen to focus on well-known

Miro-dependent mitochondrial functions to be able to understand the causality of Miro-dependent mitochondrial phenotypes. We think this is an important question and can help distinguishing primary from secondary effects of Miro loss of function. In this regard, we show that:
(i) collapse of the mitochondrial network in S2R+ cells is a rapid (and likely primary) effect of Split-Miro photocleavage;
(ii) Split-Miro-induced collapse of the mitochondrial network does not impinge on mitochondrial functionality, with regards to calcium uptake and membrane potential;
(iii) Miro is sufficient for the activation of bidirectional transport in the processes of S2R+ cells and, within the same experimental series, we show independence of Miro with regard to the processivity of mitochondrial motility. This finding was attainable only using a tool like Split-Miro which allowed us to observe the activation of mitochondrial motility (via Split-Miro overexpression) and the Miro-independence of motile properties such as velocity and run length (via Split-Miro cleavage) within the same experimental series;
(vi) mitochondrial calcium buffering is not affected by Split-Miro photocleavage, indicating that mitochondrial calcium homeostasis might not be directly controlled by Miro in S2R+ cells;
(v) Miro has a novel function in controlling fly locomotor behaviour in real time establishing a novel setup for opto-behavioural studies;
(vi) expression of human syntaphilin suppresses the effect of Miro inactivation in S2R+ cells and in adult animals.

In addition to these new findings, we have now performed additional experiments and added new data in response to the reviewers' comments which further advance our understanding of Miro functions in *Drosophila* S2R+ cells and *in vivo* (Fig. 1, 4, 5, 6, S1, S3, S5).

The authors focus on microtubule dependent transport but Miro has also been proposed to link mitochondria to Myosin motors. What are the consequences of acute Miro disruption for actin and myo19-dependent roles of Miro and mitochondrial dynamics (e.g. in vivo).

Although we have purposedly chosen to focus on well-known Miro-dependent mitochondrial functions in *Drosophila*, we recognise that actin plays an important role in influencing mitochondrial dynamics. We contemplate the notion that Miro can link mitochondria to myosin motors, and we cite two studies showing Miro-dependent mitochondrial recruitment of Myosin-19 in non-*Drosophila* cells. We now add two more references to make this point stronger (line 545).
Myosin-19 is not conserved in *Drosophila* (Odronitz et al. 2007, Genome Biol, PMID:17877792; Quintero et al. 2009, Curr Biol, PMID:9932026) and, although Miro might still recruit other myosins onto the mitochondria, it would be challenging to directly test myosin-dependent roles of Miro on mitochondrial dynamics at this stage. Previous work carried out in embryo-derived *Drosophila* cultured neurons showed that dsRNA of Myosin V and, to a lesser extent, Myosin VI, affects mitochondrial trafficking (Pathak et al. 2010, J Neurosc, PMID: 20592219). It is not known whether these myosins directly bind to Miro and, although we agree it is an interesting idea, testing this speculative hypothesis would require further long-term studies, which we believe is beyond the scope of our current article.

Quantification (according to methods) reveals the extent of Miro release into the cytoplasm. The data in Figure 1 therefore needs further quantification e.g. of the loss/gain of Miro N-term fluorescence on the mitochondria (presented as Fig1A-C). It seems important to know the dynamics of Miro remaining on the mitochondria upon phtocleavage - not just what is cytoplasmically released.

This point was also raised by another reviewer and we regret the lack of clarity regarding the tagging strategy of Split-Miro. Our aim was to show the versatility, along with the efficiency, of the Split-Miro system by using different combinations of tags. To clarify our approach, we have simplified and modified the previous Fig. 1D by adding the MitoTraker staining to show the extent of release and reassociation of mCherry-Split-MiroN. We also changed the colour-code of the image so that the re-distribution of mCherry-MiroN can be better appreciated. This is now Figure S1B-E.

At the same time, we recognise that the efficiency of dissociation and reconstitution of double-tagged Split-Miro might be different than if tagged only with one fluorophore. As suggested by the reviewer, we therefore have now expanded this part by also performing a new set of experiments in which we quantify the release and reassociation of the mCherry-Split-MiroN using a double-tagged Split-Miro (i.e. mCherry-Split-Miro-EGFP), including data showing the relative proportion of mCherry-Split-MiroN becoming cytosolic and remaining associated with the mitochondria after photocleavage, and the proportion of mCherry-Split-MiroN reassociating at the mitochondria to reconstitute Split-Miro. This is now detailed in Fig. 1C-G. We found the kinetics of dissociation and reconstitution of mCherry-Split-Miro-EGFP to be very similar to the kinetics of mCherry-Split-Miro and we observe near-complete Split-Miro dissociation and reconstitution at the mitochondria. We thank the reviewer for the opportunity to clarify this aspect of our Split-Miro design.

It's somewhat unclear what is novel in Figure 2. This seems to be mostly over expression data and numerous studies have already looked at the impact of mitochondrial trafficking of Miro up regulation. Moreover, it is unclear if this is a physiological relevant cell type to add significant insight. While the data do provide further support that split Miro behaves in a similar way to Miro it does not provide much new insight on the importance of miro for regulating mitochondrial trafficking.

We regret not making clearer what the new information provided in Fig. 2 is and the importance of using an overexpression system in our experimental setup.
As part of the characterisation of our novel tool, it is crucial to demonstrate that Split-Miro behaves like Miro in over-expression. At the same time, the experiments described in Fig. 2 (and related S2 Fig) show that, although mitochondrial trafficking is upregulated after Split-Miro overexpression (Fig. 2F-G, H), Split-Miro photocleavage did not have any major effects on the velocity and run length of the moving organelles, which remained elevated after Split-Miro over-expression and did not return to control levels (Fig. 2H and S2J-K Fig). These results indicate that the proportion and processivity of motile mitochondria are controlled by separate Miro-dependent and Miro-independent mechanisms, respectively, and suggest Miro has a dual role in regulating mitochondrial motility (Fig. 2I). These findings could be obtained thanks to the specific setup that we designed with combined upregulation (via overexpression) and downregulation (via Split-Miro photocleavage). We now specify this in the text adding in lines 232-233, *"[…] using a strategy combining upregulation (via overexpression) and downregulation (via Split-Miro photocleavage) […]"*.

Although the over-expression system is essential in our experimental setup, we also performed experiments in cellular backgrounds where Miro abundance is reduced by RNAi. By doing so, we were able to confirm the key results of unchanged velocity (S2K Fig) after photocleavage, as well as the mitochondrial network retraction phenotype. In addition, we manipulate the behaviour of adult animals by overexpressing Split-Miro with neuronal specificity in Miro KO animals (Fig. 7), which is a considerable feat. Our new *in vivo* data (Fig. 5, 6) are also generated in *miro*$^{+/-}$ genetic backgrounds that have reduced Miro levels.

With regards to the point of S2 cells being a relevant model system, we note that this cell type has been used extensively for cell biological studies in *Drosophila* and have yielded important discoveries for the trafficking field. We added key references in the manuscript (lines 123-125) to support this notion.

Can the authors present examples of the presumably highly artificial 'processes' induced by cytochalasin D treatment? These processes apparently have microtubules of uniform polarity (e.g. plus end out) but they require actin depolymerisation for their formation. How does this impact interpretation given Miro also couples to actin transport pathways.

We have indicated with white arrows in Fig. 1A examples of processes that the S2 cells can be induced to form after treatment with cytochalasin D. This is very useful to study microtubule-based motility in this cell type and this treatment has been used by other groups for this purpose. We cite these studies and we make clear in the manuscript that we are focusing on microtubule-based motility. We also notice that new important work for the field has focused on the microtubule-rich processes of S2 cell (see, for example, Ventura 2023 https://www.biorxiv.org/content/10.1101/2023.03.31.535077v1). Nonetheless, in the original manuscript we acknowledge that the actin is mostly depolymerised in this experimental setup (S6B Fig) and we have provided evidence that our dynamic mitochondrial retraction phenotype following Split-Miro photocleavage is not affected by the presence of actin (S6A Fig).

The speed at which mitochondrial rearrangement occurs is is certainly intriguing but the overall phenotype of light induced Miro loss of function is very reminiscent of what has been shown previously upon Miro loss of function or upon calcium-dependent mitochondrial uncoupling from microtubules. ie collapsed mitochondrial networks with shorter mitochondria. The authors do not sufficiently articulate what major novel finding they achieve using the new optogenetic approach.

We agree with the reviewer that an effect on mitochondrial morphology deriving from Miro loss of function is well-known and evolutionary conserved from yeast to mammals, and we cite the relevant literature in our manuscript. We have purposedly chosen to focus on well-known Miro-dependent mitochondrial functions to be able to understand the causality of Miro-dependent mitochondrial phenotypes. We think this is an important question and can help distinguishing primary from secondary effects of Miro loss of function. However, specifically to the point raised by the reviewer, we show that collapse of the mitochondrial network in S2R+ cells is a rapid (and likely primary) effect of Miro loss of function, which was unknown. It is important to highlight that, owing to the rapid nature of our manipulation, we are able to capture the dynamic collapse of the network that is likely to contribute to the latter reduction of the mitochondria from the cell processes. We make this point in the manuscript (lines 257-260).
Furthermore, despite the known role of Miro in maintaining mitochondrial morphology, it was not known whether this was due to a tethering effect onto other cellular structures or a direct role on the mitochondrial membrane. Using a Miro-independent mechanism by overexpressing SNPH, we have on one hand demonstrated that the morphological transition depends on, and can be prevented by, microtubule tethering. On the other hand, we show that mitochondrial motility might be dispensable for mitochondrial calcium homeostasis, at least within the rapid timeframe of our experimental setup. We think these are important conceptual advances.

Calcium-dependent mitochondrial remodelling (for example, Nemani et al., Cell Reports, 2017, PMID: 29694881) was proposed to lead to potential, but undemonstrated, uncoupling from the microtubules. We show a different mechanism in that Miro-

dependent mitochondrial release from the microtubules leads to a drastic morphological change of the mitochondrial network, independently of calcium.
What we find remarkable is that, even if the Miro-dependent mitochondria remodelling is significant, mitochondrial functionality is retained, at least with regards to the mitochondrial calcium uptake and membrane potential and within the rapid timeframe of our experiments. This is an area of active research for our lab going forward.

Moreover from the movies it looks like large amounts of trafficking remain upon photocleavage? Can the authors explain this? What underlies remaining movement?? Is this microtubule dependent?
It is known that microtubule-dependent mitochondrial transport does not depend entirely on Miro, we discuss this aspect in depth in our manuscript, and so it is not surprising that there is still movement, likely microtubule-based, after Miro photocleavage. We now also provide evidence this is the case *in vivo* (Fig. 5). Thus, with our manipulations we are not abolishing transport completely, rather we are upregulating and downregulating transport.

The authors mention that there is no effect on peroxisome distribution but what about trafficking kinetics of these organelles?
We have now quantified the duty cycle of peroxisomes in wt-Miro and Split-Miro backgrounds and added this data to S3C Fig. Contrarily to what observed for mitochondrial trafficking, we see no difference between wt-Miro and Split-Miro after blue-light irradiation.

It is also somewhat surprising that the authors do not further investigate the impact of Miro cleavage on mitochondrial interactions with the ER. it seems a significantly missed opportunity to explore these aspects given that much of what the authors demonstrate with split miro is confirmatory of what is already known about Miro function.
We agree with the reviewer that, similarly to the other Miro-dependent functions that we have analysed in our manuscript, Miro has a documented role at the mitochondria-ER interface in mammalian cultured cells (COS7 and MEF cells) as well as in *Drosophila*. We mention this possibility in our manuscript citing the relevant mammalian literature (lines 324-327) and we now add here also the *Drosophila* reference (Lee 2016 Dev Cell; PMID: 27093086) otherwise cited elsewhere. Therefore, there is scope to interrogate whether maintaining mitochondria-ER association may be a primary role of Miro, and we used Split-Miro to address this question.
We first produced a new construct for expression in S2 cells to mark mitochondria-ER contact sites by split-GFP. This sensor is based on SPLICS technology (Cieri 2017, Hewitt 2022, Granat 2022, cited in our manuscript) and allows the visualisation of mitochondria-ER contact sites by live imaging. We then transfected S2R+ cells with the SPLICS construct and quantified the effect of Split-Miro photocleavage on the number of contact sites. We found no significant effect induced by Split-Miro. Because we think this is an important point raised by the reviewer, we added this experiment in the main Fig. 4 as it is relevant to the mitochondrial calcium uptake experiments and modified the discussion as well as a result. We thank the reviewer for their suggestion which also strengthens the case that mitochondrial calcium uptake, which is known to be affected by mitochondria-ER association, is not a primary role of Miro in this context.

Although the rescue by syntaphalin is potentially interesting several of the images in Fig 3 e.g. E, E' are of poor resolution and make it hard to see what is presented in the qualified data.
With this experiment we wanted to combine optogenetic stimulation with three-colour imaging and fast acquisition using a laser-scanning confocal. In addition to this, we use low laser power to reduce photobleaching of the fluorophores, including the vital dye MitoTracker DeepRed (MTDR). We have now expanded our detailed Methods

section to make our point clearer (lines 717-718). Because of the complex imaging setup, with specific microscope filter settings and fluorophore combination, the overall staining may appear 'grainer', especially in enlargements of smaller areas such as Fig. 3E'. We do not think this is abnormal and we are confident the quality of our data is adequate to quantify the clear phenotype of mitochondrial retraction in the presence of Split-Miro and the rescue of this phenotype by SNPH. Instead, using the MTDR stain, which marks the intra-mitochondrial space instead of the outer mitochondrial membrane like Miro and Split-Miro, gives us further confidence about the robustness of the Split-Miro-dependent phenotypes, irrespective of how the mitochondria are visualised.
We also notice that one of the first reports to simultaneously image three distinct sub-mitochondrial structures in living cells using optical nanoscopy and MitoTracker Deep Red (Opstad 2018, Nanophotonics, https://doi.org/10.1515/nanoph-2017-0112) show that the quality of the resolution inversely correlates with the number of probes used for imaging. Even at super-resolution level (and without the optogenetic stimulation protocol), the three-colour images with vital dyes do not appear of a significantly better resolution than ours.

The authors demonstrate that Miro loss of function does not disrupt calcium entry into mitochondria. This does not seem particularly surprising given than the main calcium entry pathway is through MCU on the IMM. Miro has been shown to form complexes with MCU but at least in mammalian cells mitochondria lacking Miro can still take up calcium.
While in *Drosophila* tissues lack of Miro has a reported effect on mitochondrial calcium homeostasis (Lee et al. Dev Cell 2016, PMID: 27093086; Li et al. Nat Metab 2021, PMID: 34504353), our understanding is that there is less consensus about the role of Miro in mitochondrial calcium homeostasis in mammals (as discussed in Nguyen 2014, PNAS; PMID: 25136135), potentially due to the presence of two functionally distinct Miro orthologs, Miro1 and Miro2 (Lopez-Domenec 2018, EMBO J; PMID: 29311115). While there is evidence suggesting lack of Miro1 does not affect $[Ca]_m$ uptake in MEFs cells (Nguyen 2014, PNAS), mutation of the Miro1 EF-hands alters $[Ca]_m$ uptake in primary mouse hippocampal neurons (Chang 2011, PNAS; PMID: 21876166), as it does overexpression of Miro1 in primary rat cortical neurons (Saotome 2008, PNAS; PMID: 19098100).
We cannot exclude, however, that the transmembrane domain of Miro (which likely binds to MCU in mammals) could somehow still retain some functionality after Split-Miro photocleavage and affect calcium uptake in the mitochondria, as also suggested by another reviewer. We have now modified the discussion to make this point clearer (lines 560-563).

The in vivo experiments provide potential interesting findings supporting an important role for Miro in animal physiology. But there is currently insufficient data reporting the impact of Miro cleavage on the mitochondrial network in vivo or what the cellular consequences. As such it remains unclear how photocleavage leads to the altered physiology.
We agree with the reviewer that more *in vivo* evidence of the effects of Split-Miro would strengthen the manuscript. We have now performed a new set of experiments by generating new genetic backgrounds and show that Split-Miro impacts on neuronal activity and mitochondrial trafficking in live flies (Fig. 5 and 6).

The neuronal activity data (Fig. 5) that we provide are consistent with previous studies showing that "*[…] reducing mitochondrial number positively correlates with activity-dependent vesicular release at the presynapses […]*". These data also support the notion that Split-Miro could affect fly behaviour by triggering an imbalance in neuronal activity, as speculated in our original discussion: '*It is conceivable that Split-Miro triggers an acute imbalance of neuronal homeostasis and synaptic transmission, which leads to augmented locomotor activity*'.

The *in vivo* trafficking data show Split-Miro-dependent reduced mitochondrial trafficking in 2-day-old flies, but not in older flies (Fig. 5 and S5E-H Fig), when transport is known to decline in this system (Vagnoni et al., 2016, PMID: 26598558; Vagnoni and Bullock, 2018, PMID: 29606421). We find this dataset very interesting: on one hand it strengthens the case for co-existence of Miro-dependent vs Miro-independent transport, as argued with the work done in S2R+ cells. On the other hand, these new data put forward an interesting scenario whereby while Miro is crucial for mitochondrial transport in younger flies, the remaining mitochondrial trafficking in aged flies is largely Miro-independent and could be accounted for by other mechanisms. We have now changed large parts of the result and discussion sections to integrate these new findings.

Movies: The authors should provide a similar example movie imaging mitochondria under identical photo illumination conditions in cells not over expressing Miro or expressing WT-Miro to demonstrate that the illumination conditions used to disrupt split miro do not impact mitochondrial trafficking.
We have now provided this additional movie as Supplementary Movie 5.

The example movies provided (e.g. Figure 2) reveal that upon photo cleavage parts of the mitochondrial network remodel. However it also clear that large amounts of bi-directional trafficking of mitochondria remain throughout the movie.
It is known that microtubule-dependent mitochondrial transport does not dependent entirely on Miro, which we discuss extensively throughout the manuscript, and so it is not surprising that there is still movement, likely microtubule-based, after Miro photocleavage. Thus, with our manipulations we are not abolishing transport completely, rather we are upregulating (via Miro and Split-Miro overexpression) and downregulating transport (via Split-Miro photocleavage).
